# Trends of infant mortality and its determinants in Ethiopia: mixed-effect binary logistic regression and multivariate decomposition analysis

**DOI:** 10.1186/s12884-021-03835-0

**Published:** 2021-05-05

**Authors:** Getayeneh Antehunegn Tesema, Wullo Sisay Seretew, Misganaw Gebrie Worku, Dessie Abebaw Angaw

**Affiliations:** 1 Department of Epidemiology and Biostatistics, institute of public health, college of medicine and health science, University of Gondar, Gondar, Ethiopia; 2 Department of Human Anatomy, School of Medicine, College of Medicine and Health Science, University of Gondar, Gondar, Ethiopia

**Keywords:** Infant mortality, Mixed effect analysis, Multivariate decomposition analysis, Ethiopia

## Abstract

**Background:**

Infant mortality remains a serious global public health problem. The global infant mortality rate has decreased significantly over time, but the rate of decline in most African countries, including Ethiopia, is far below the rate expected to meet the SDG targets. Therefore, this study aimed to investigate the trends of infant mortality and its determinants in Ethiopia based on the four consecutive Ethiopian Demographic and Health Surveys (EDHSs).

**Methods:**

This analysis was based on the data from four EDHSs (EDHS 2000, 2005, 2011, and 2016). A total weighted sample of 46,317 live births was included for the final analysis. The logit-based multivariate decomposition analysis was used to identify significantly contributing factors for the decrease in infant mortality in Ethiopia over the last 16 years. To identify determinants, a mixed-effect logistic regression model was fitted. The Intra-class Correlation Coefficient (ICC) and Likelihood Ratio (LR) test were used to assess the presence of a significant clustering effect. Deviance, Akaike Information Criteria (AIC), and Bayesian Information Criteria (BIC) were used for model comparison. Variables with a *p*-value of less than 0.2 in the bi-variable analysis were considered for the multivariable analysis. In the multivariable analysis, the Adjusted Odds Ratio (AOR) with 95% Confidence Interval (CI) were reported to identify the statistically significant determinants of infant mortality.

**Results:**

Infant mortality rate has decreased from 96.9 per 1000 births in 2000 to 48 per 1000 births in 2016, with an annual rate of reduction of 4.2%. According to the logit based multivariate decomposition analysis, about 18.1% of the overall decrease in infant mortality was due to the difference in composition of the respondents with respect to residence, maternal age, type of birth, and parity across the surveys, while the remaining 81.9% was due to the difference in the effect of residence, parity, type of birth and parity across the surveys. In the mixed-effect binary logistic regression analysis; preceding interval <  24 months (AOR = 1.79, 95% CI; 1.46, 2.19), small size at birth (AOR = 1.55, 95% CI; 1.25, 1.92), large size at birth (AOR = 1.26, 95% CI; 1.01, 1.57), BMI <  18.5 kg/m^2^ (AOR = 1.22, 95% CI; 1.05, 1.50), and twins (AOR = 4.25, 95% CI; 3.01, 6.01), parity> 6 (1.51, 95% CI; 1.01, 2.26), maternal age and male sex (AOR = 1.50, 95% CI: 1.25, 1.79) were significantly associated with increased odds of infant mortality.

**Conclusion:**

This study found that the infant mortality rate has declined over time in Ethiopia since 2000. Preceding birth interval, child-size at birth, BMI, type of birth, parity, maternal age, and sex of child were significant predictors of infant mortality. Public health programs aimed at rural communities, and multiparous mothers through enhancing health facility delivery would help maintain Ethiopia’s declining infant mortality rate. Furthermore, improving the use of ANC services and maternal nutrition is crucial to reducing infant mortality and achieving the SDG targets in Ethiopia.

## Background

The death of a child during the first year of life is called infant mortality [1]. The global infant mortality has fallen dramatically from 8.8 million in the last two decades to 4.1 million [2] and the rate dropped from 65 deaths per 1000 live births in 1990 to 29 deaths per 1000 live births in 2017 [3]. Despite the substantial decline in global infant mortality, low-and middle-income countries still bear the enormous burden of infant mortality [2, 4, 5]. Ethiopia is one of the countries with a high infant mortality rate (48 per 1000 live births) in Africa [6]. Nearly 80% of infant deaths are from preventable causes [7].

Infant mortality is one of the commonest health-related indicators used to assess the health status of the community [8]. Despite the progress made by many countries to achieve the Millennium Development Goal (MDG) 4 to reduce child mortality by two-thirds [9]; half of the world’s nations including Ethiopia are still behind their targets and MDG-4 continued as unfinished agenda [10].

The Ethiopian government strongly motivated to improve maternal-child and maternal health for the last two decades [11] but infant mortality remains a significant health care problem in the country [12]. It has reduced from 123 per 1000 births in 1990 to 48 per 1000 births in 2015 but it is far below the national target [13, 14]. According to the Ethiopian Demographic and Health Surveys (EDHSs), the infant mortality rate has declined from 97 per 1000 live births in 2000 [15] to 48 per 1000 live births in 2016 [6] with a huge disparity across regions and within countries [12].

Maternal age [16, 17], maternal education status [18, 19], household wealth status [20], Antenatal Care (ANC) visit during pregnancy [21, 22], parity [23], birth order [24], place of delivery [25, 26], child nutritional status (stunting, wasting and underweight) [26], vaccination status [27], and residence [28] were reported by previous researchers as significant predictors of infant mortality.

Though infant mortality rates have decreased over time in Ethiopia, previous studies were focused on the prevalence and associated factors of infant mortality only [21, 29, 30] and failed to capture the trends and determinants of infant mortality in Ethiopia over time using a Logit based Multivariate Decomposition analysis for Non-linear Response Model (MVDCMP) and Generalized Linear Mixed Model (GLLM). Therefore, this study aimed to investigate the trend and determinants of infant mortality in Ethiopia over time. Understanding the trends and determinants of infant mortality could help public health planners, and partners to design evidence-based interventions to effectively reduce infant mortality in Ethiopia.

## Methods and materials

### Data sources

A community-based time-series cross-sectional study was used to answer the research objectives. All the Demographic and Health Surveys (DHSs) (EDHS 2000, 2005, 2011, and 2016) conducted in Ethiopia were used. The EDHS was employed in every five-year interval to generate updated health and health-related indicators. The majority of the country’s population lives in the regional states of Amhara, Oromia, and Southern Nations Nationalities and People’s Regions (SNNPR) [25]. Ethiopia is the 13th in the world and 2nd most populous country in Africa [26]. In 2016, there were an estimated 102 million people.

A two-stage sampling technique was employed to select the sample and a total of 539 Enumeration Areas (EAs) in EDHS 2000, 540 EAs in EDHS 2005, 624 EAs in EDHS 2011, and 645 EAs in EDHS 2016 were randomly selected. Then, on average 27 to 32 households per EA were selected. The source population was all live births from reproductive-age women within 5 years before the survey in Ethiopia whereas all live births from reproductive-age women in the selected enumeration areas were the study population. A total weighted sample of 46,317 live births (12,260 in EDHS 2000, 11,163 in EDHS 2005, 11,872 in EDHS 2011, and 11,022 in EDHS 2016) from reproductive-age women were used for analysis. The detailed sampling procedure was presented in the full EDHSs report [18, 19, 31, 32].

### Study variables

The outcome variable for this study was infant mortality (the death of live birth within 1 year of birth). In EDHS there was a question about whether the child was alive or died at the time of the survey and for dead infants-age at death were recorded. Death of a child within 1 year of age was coded as 1, and 0 if the child was alive. The unit of analysis in this study was all live births in the 5 years preceding the survey. The infant mortality rate is defined as the number of infant deaths per 1000 live births [33]. The independent variables considered in this study were region (coded as Tigray, Afar, Amhara, Oromia, Somali, Benishangul, SNNPR, Gambella, Harari, Addis Ababa, and Dire Dawa), residence (coded as rural, and urban), sex of household head (coded as male and female), maternal age (recoded as < 20, 20–29, 30–39 and 40–49 years), women education (recoded as no, primary, and secondary and higher), paternal education (recoded as no, primary, and secondary and above), preceding birth interval (recoded as < 24 and ≥ 24 months), Body Mass Index (BMI) of the mother (recoded as < 18.5, 18.5–24.9, and ≥ 25 kg/m^2^), wealth index (coded as poor, middle and rich), parity (recoded as 1–3, 4–6 and >  6 births), type of birth (coded as single and multiple), place of delivery (coded as home and health facility), ANC visit during pregnancy (no visit, 1–4 and > 4 ANC visits), cigarettes smoking (coded as no and yes), mode of delivery (coded as vaginal and caesarean delivery), child nutritional status (stunting; coded as normal, moderately stunted, and severely stunted; wasting coded as normal, moderately wasted, and severely stunted; and underweight coded as normal, moderately underweight and severely underweight), media exposure (coded as no and yes), religion (coded as orthodox, muslim, protestant, catholic and others), sex of child (coded as male and female), covered by health insurance (coded as no and yes), and birth weight (large, average and small).

Wealth Index (WI) was considered as a living standard measure for each respective year and generated using the Principal Component Analyses (PCA). The variables included in the PCA were ownership of durable assets, like radios, cars, refrigerators, TV sets, motorcycles, and bicycles; housing characteristics, such as the number of rooms for sleeping and building materials (walls, floors, and roofs); access to utilities and infrastructures, like electricity supply, source of drinking water, and sanitation facilities.

### Data management and analysis

The Ethiopian Demographic and Health survey consists of different datasets including men, women, kids (KR), birth, household, and household datasets. For this study, we used the Kids Record (KR) data set. The data were weighted using sampling weight, primary sampling unit, and strata before any statistical analysis to restore the representativeness of the survey to get reliable statistical estimates. Descriptive and summary statistics were done using STATA version 14 software.

### Trend analysis

For the decomposition analysis, we appended the extracted data of 2000, 2005, 2011 and 2016 using the STATA command “append using” after we kept the similar variables across the surveys. The change in infant mortality rate in Ethiopia for the last 16 years was examined.

To determine the factors that contributed to the decrease in the infant mortality rate over the last 16 years, the Multivariate Decomposition Analysis for the Non-linear Response variable (MVDCMP) was used. The multivariate decomposition analysis based on the logit link function uses the output from the binary logistic regression model to divide into components. The decrease in infant mortality can be explained by the difference in composition between the surveys (i.e., differences in characteristics or endowment) and/or the difference in effects of the explanatory variables across the surveys (i.e., differences in coefficients).

The multivariate decomposition analysis of the logit or log-odd of infant mortality is taken as:

$$ {\displaystyle \begin{array}{l}\mathrm{Logit}\ \left(\mathrm{A}\right)-\mathrm{Logit}\ \left(\mathrm{B}\right)=\mathrm{F}\ \left(\mathrm{XA}\upbeta \mathrm{A}\right)-\mathrm{F}\ \left(\mathrm{XB}\upbeta \mathrm{B}\right)\\ {}\frac{=\left[\mathrm{F}\ \left(\mathrm{XA}\upbeta \mathrm{A}\right)-\mathrm{F}\ \left(\mathrm{XB}\upbeta \mathrm{A}\right)\right]}{\mathrm{E}}+\frac{\Big[\mathrm{F}\ \left(\mathrm{XB}\upbeta \mathrm{A}\right)-\mathrm{F}\ \left(\mathrm{XB}\upbeta \mathrm{B}\right]}{\mathrm{C}}\end{array}} $$

The E component refers to the part of the overall decrease in infant mortality explained by the change in the composition of the study participants across the surveys. There is no error term in the logit-based multivariate decomposition analysis because we used the logit link function. The C component refers to the percentage of the overall decrease in infant mortality attributable to the differences in coefficients or effects of the explanatory variable across the surveys. For the decomposition analysis of infant mortality using the mvdcmp STATA command (28). Variables with a *p*-value < 0.2 in the bi-variable Logit-based multivariate decomposition analysis were considered for the multivariable Logit-based multivariate decomposition analysis. Finally, *p*-value < 0.05 and the corresponding coefficient (*B*) with a 95% confidence interval were used to declare significant factors that contributed to the decrease in infant mortality.

### Determinants of infant mortality

As the data used for this study had nested structure, infants within the same cluster might share similar characteristics than infants out of that cluster. In hierarchical data, advanced statistical models such as mixed-effect regression analysis to get reliable estimate. Therefore, a two-level mixed-effect logistic regression model (both fixed and random effect) was fitted using EAs as a random variable to draw a valid conclusion. The assumptions of the mixed-effect binary logistic regression model were checked using the Intra-class Correlation Coefficient (ICC) and Likelihood Ratio (LR) test. The Median Odds Ratio (MOR) and Proportional Change in Variance (PCV) were computed to measure the variation across clusters. ICC quantifies the degree of heterogeneity of infant mortality between clusters (the proportion of the total observed individual-level variation in infant mortality that is attributable to between cluster variations).

$$ {\displaystyle \begin{array}{l}\\ {}\mathrm{ICC}=\upsigma 2/\left(\upsigma 2+\uppi 2/3\right),\end{array}} $$

The MOR measures the between cluster variation in terms of odds ratio. The median value of the odds ratio between the cluster at high risk of infant mortality and cluster at lower risk of the infant when randomly picking out two clusters (EAs).

$$ \mathrm{MOR}=\exp\ \left(\surd \left(2\ast \partial 2\ast 0.6745\right)\right)\sim \mathrm{MOR}=\exp\ \left(0.95\ast \partial \right) $$

∂2 indicates that cluster variance

PCV measures the total variation in infant mortality explained by the final model compared to the null model.

$$ \mathrm{PCV}=\left(\operatorname{var}\ \left(\mathrm{null}\ \mathrm{model}\right)-\operatorname{var}\ \left(\mathrm{full}\ \mathrm{model}\right)\right)/\left(\operatorname{var}\ \left(\mathrm{null}\ \mathrm{model}\right)\right) $$

Akaike Information Criteria (AIC), Bayesian Information Criteria (BIC), and deviance were used for model comparison and a model with the lower deviance was chosen since the model was nested. We identified the independent variables based on previous literature conducted on determinants of infant mortality. As the data used for this study was secondary there was missing on the outcome variable (age at death), and we drop the observation that has missing value on the outcome variables. In the bi-variable mixed-effect binary logistic regression analysis; residence, sex of household head, maternal age, maternal education, wealth status, maternal BMI, preceding birth interval, parity, covered by health insurance, size at birth, ANC visit during pregnancy, sex of the child, place of delivery and type of birth had a *p*-value less than 0.2 and were considered for multivariable analysis. However, in the multivariable analysis; parity, type of birth, maternal age, maternal BMI, number of ANC visits, preceding birth interval, sex of a child, and size at birth were significantly associated with infant mortality. The Adjusted Odds Ratio (AOR) with a 95% Confidence Interval (CI) and *p*-value < 0.05 in the multivariable model were used to declare significant determinant factors of infant mortality.

### Ethical consideration

As the study was a secondary data analysis accessed from the MEASURE DHS program, this study did not require ethical approval and participant consent. We have granted permission from http:/www.dhsprogram.com to download and use the data for this study. In the data sets, there is no name of persons or household addresses.

## Results

### Characteristics of the study population

A total of 46,317 live births were included in this study. The non-response rate in EDHS 2000, EDHS 2005, EDHS 2011, and EDHS 2016 were 2.2, 4, 5 and 5%, respectively. More than one-third of live births in all four surveys were found in the Oromia region. The proportion of mothers who had a primary level of education slightly increased from 13% in 2000 to 26.8% in 2016. Besides, the percentage of women who had media exposure has increased from 27.1 to 33.1% in the last 16 years. Regarding ANC visits during pregnancy, the percentage of women who had 1–4 ANC visits during pregnancy was increased from 19.8% in 2000 to 46.6% in 2016. The percentage of health facility delivery has increased from 5.2 to 27.4% for the last 16 years (Table 1).

**Table 1:** Characteristics of the study population in 2000, 2005, 2011 and 2016 Ethiopian Demographic and Health Surveys

Variables	EDHS 2000 (*n* = 12,260)	EDHS 2005 (*n* = 11,163)	EDHS 2011(*n* = 11,872)	EDHS 2016 (*n* = 11,022)
	Weighted frequency (%)	Weighted frequency (%)	Weighted frequency (%)	Weighted frequency (%)
**Region**				
Tigray	788 (6.4)	698 (6.3)	753 (6.3)	716 (6.5)
Afar	126 (1.0)	107 (1.0)	121 (1.0)	114 (1.0)
Amhara	3202 (26.1)	2621 (23.5)	2656 (22.4)	2072 (18.8)
Oromia	4999 (40.9)	4411 (39.5)	5014 (42.2)	4851 (44.2)
Somali	142 (1.2)	477 (4.3)	364 (3.1)	507 (4.6)
Benishangul	124 (1.0)	105 (0.9)	140 (1.2)	122 (1.1)
SNNPRs	2602 (21.2)	2500 (22.4)	2494 (21.1)	2296 (20.8)
Gambella	29 (0.2)	31 (0.3)	40 (0.3)	27 (0.2)
Harari	25 (0.2)	22 (0.2)	29 (0.2)	26 (0.2)
Addis Ababa	182 (1.5)	153 (1.3)	221 (1.9)	244 (2.2)
Dire Dawa	40 (0.3)	37 (0.3)	39 (0.3)	47 (0.4)
**Place of residence**				
Urban	1276 (10.4)	815 (7.3)	1528 (12.9)	1215 (11.0)
Rural	10,984 (89.6)	10,348 (92.7)	10,344 (87.1)	9807 (89.0)
**Religion**				
Orthodox	6042 (49.3)	4674 (41.9)	4519 (38.1)	3772 (34.2)
Catholic	81 (0.7)	121 (1.1)	108 (0.9)	103 (0.9)
Protestant	1959 (16.0)	2217 (19.9)	2758 (23.2)	2329 (21.1)
Muslim	3713 (30.3)	3875 (34.7)	4214 (35.5)	4561 (41.4)
Others	465 (3.8)	275 (2.5)	271 (2.3)	257 (2.3)
**Age of women (in years)**				
< 20	557 (4.6)	575 (5.2)	491 (4.1)	378 (3.4)
20–29	6047(49.3)	5415 (48.5)	6158 (51.9)	5421 (49.2)
30–39	4272 (34.8)	4019 (36.0)	4166 (35.1)	4261 (38.7)
≥ 40	1384 (11.3)	1154 (10.3)	1057 (8.9)	962 (8.7)
**Maternal education**				
No education	10,062 (82.1)	8838 (79.2)	8227 (69.3)	7284 (66.1)
Primary	1597 (13.0)	1855 (16.6)	3211 (27.0)	2950 (26.8)
Secondary and higher	601 (4.9)	470 (4.2)	434 (3.7)	788 (7.1)
**Husband education**				
No	7771 (63.4)	6508 (58.3)	4866 (47.0)	4116 (37.3)
Primary	3026 (24.7)	3350 (30.0)	583 (4.9)	798 (7.30
Secondary and higher	1463 (11.9)	1305 (11.7)	6422 (54.1)	6108 (55.4)
**Sex of household head**				
Male	10,589 (86.4)	9943 (89.1)	10,106 (85.1)	9494 (86.1)
Female	1671 (13.6)	1220 (10.9)	1766 (14.9)	1528 (13.9)
**Maternal BMI (in Kg/m**^**2**^**)**				
< 18.5	2764 (22.5)	1109 (9.9)	2439 (20.6)	2098 (19.0)
18.5–24.9	9112 (74.3)	4146 (37.1)	8678 (73.1)	7953 (72.2)
≥ 25	384 (3.1)	5908 (53.0)	755 (6.3)	971 (8.8)
**Number of ANC visit during pregnancy**				
No visit	5789 (72.7)	5225 (46.8)	4516 (57.1)	2818 (37.1)
1–4 visits	1573 (19.8)	4447 (39.8)	2451 (31.0)	3536 (46.6)
> 4 visits	604 (7.5)	1491 (13.4)	940 (1.9)	1236 (16.3)
**Child size at birth**				
Small	4115 (33.6)	3557 (31.9)	3519 (29.6)	2957 (26.8)
Average	4376 (35.7)	4462 (40.0)	4548 (38.3)	4580 (41.6)
Large	3769 (30.7)	3144 (28.1)	3805 (32.1)	3485 (31.6)
**Place of delivery**				
Home	11,625 (94.8)	10,502 (94.1)	10,627 (89.5)	7997 (72.6)
Health facility	635 (5.2)	661 (5.9)	1245 (10.5)	3025 (27.4)
**Mode of delivery**				
Vaginal	12,160 (99.3)	11,050 (99.0)	11,697(98.5)	10,810 (98.1)
Caesarean section	100 (0.7)	113 (1.0)	175 (1.5)	212 (1.9)
**Type of birth**				
Single	11,994 (97.8)	10,963 (98.2)	11,597 (97.7)	10,730 (97.4)
Multiple	266 (2.2)	200 (1.8)	275 (2.3)	292 (2.6)
**Sex of infant**				
Male	6288 (51.3)	5723 (51.3)	6168 (52.0)	5724 (51.9)
Female	5972 (48.7)	5440 (48.7)	5704 (48.0)	5298 (48.1)
**Ever had termination of pregnancy**				
No	10,488 (85.5)	10,267 (92.0)	10,639 (89.6)	10,056 (91.2)
Yes	1772 (14.5)	896 (8.0)	1233 (10.4)	966 (8.8)
**Stunting**				
Normal	7644 (62.4)	9149 (82.0)	6893 (58.1)	6624 (60.1)
moderately stunted	2292 (18.7)	980 (8.8)	2258 (19.0)	1802 (16.3)
Severely stunted	2324 (18.9)	1034 (9.2)	2721 (22.9)	2596 (23.6)
**Underweight**				
Normal	7937 (64.7)	9487 (85.0)	7288 (61.4)	6998 (63.5)
Moderately underweight	2837 (23.1)	1192 (10.7)	2615 (22.0)	2146 (19.5)
Severely underweight	1486 (12.2)	484 (4.3)	1969 (16.6)	1879 (17.0)
**Wasting**				
Normal	11,091 (90.5)	10,701 (95.7)	9896 (83.4)	8931 (81.0)
Moderately wasted	1025 (8.3)	368 (3.5)	758 (6.4)	686 (6.2)
Severely wasted	144 (1.2)	94 (0.8)	1218 (10.2)	1406 (12.8)
**Media exposure**				
No	8932 (72.9)	7017 (62.9)	6988 (58.9)	7375 (66.9)
Yes	3328 (27.1)	4146 (37.1)	4884 (41.1)	3647 (33.1)
**Preceding birth interval**				
< 24 months	1950 (15.9)	2246 (20.1)	1963 (16.5)	1942 (17.6)
≥ 24 months	10,310 (84.1)	8917 (79.9)	9909 (83.5)	9080 (82.4)
**Parity**				
1–3	5338 (43.6)	4576 (41.0)	5295 (44.6)	4836 (43.9)
4–6	4038 (32.9)	3962 (35.5)	4014 (33.8)	3732 (33.9)
> 6	2884 (23.5)	2625 (23.5)	2564 (21.6)	2454 (22.2)

### Trends of infant mortality rate from 2000 to 2016

**Fig. 1:**
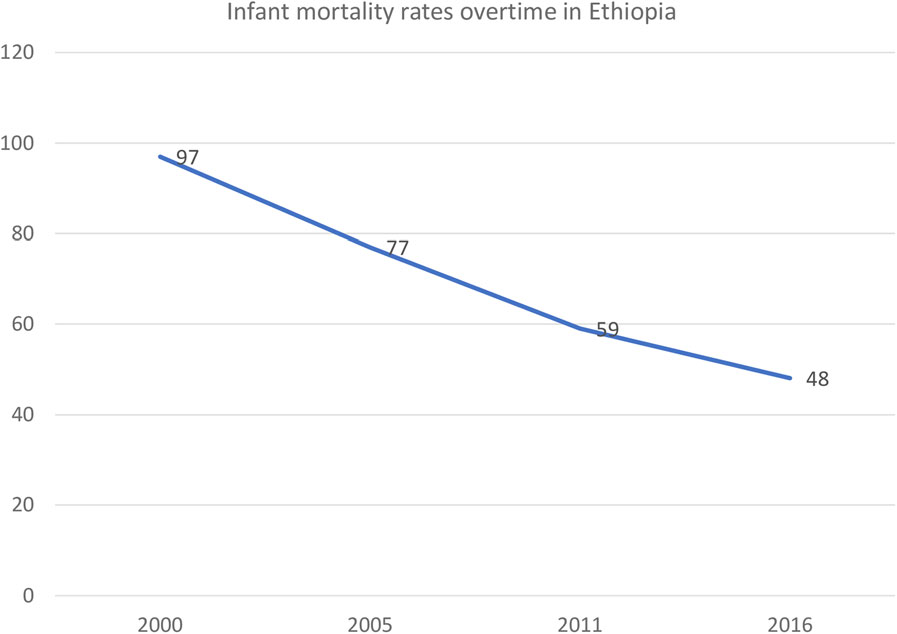
The trends of infant mortality rate among live births from reproductive-age women in Ethiopia over time (2000–2016)

**Fig. 2:**
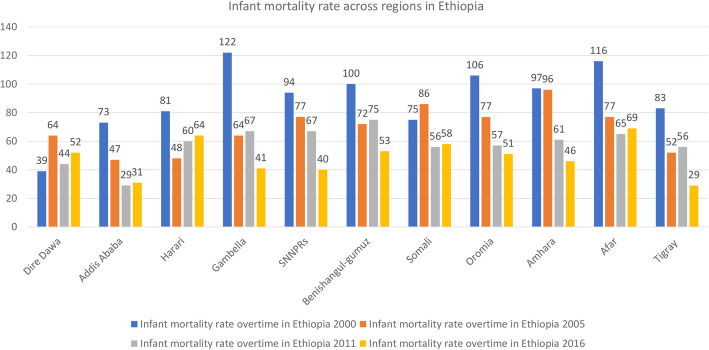
The trends of infant mortality rate across region regions in Ethiopia over time (2000–2016)

The overall infant mortality rate has decreased from 96.9 [93.6, 104.2] per 1000 live births in 2000 to 48.0 [44.2, 52.2] to 1000 live births in 2016 with an Annual Rate of Reduction (ARR) of 4.2% (Fig. 1). The infant mortality rate has decreased across regions over time, it was decreased from 122 per 1000 live births in 2000 to 41 per 1000 live births in 2016 in the Gambella region (Fig. 2). Regarding the place of residence, there was a 54.2- and 51.5-point decrease in the infant mortality rate among urban residents and mothers who had no formal education over the last 16 years, respectively. The infant mortality rate among births in the health facility decreased by 66.1 per 1000 live births from 2000 to 2016 (Table 2).

**Table 2:** Trends in infant mortality rate among births from reproductive-age women in the last five years before the surveys by selected characteristics in Ethiopia in 2000, 2005, 2011, and 2016 Ethiopia Demographic and Health Surveys

Variables	2000	2005	2011	2016	Point difference in infant mortality rate over time			
					2005–2000	2011–2005	2016–2011	2016–2000
**Place of residence**								
Urban	96.4	71.7	53.5	42.2	−24.7	−18.2	−11.3	−54.2
Rural	99.0	80.5	60.6	48.8	−18.5	−19.9	− 11.8	−50.2
**Religion**								
Orthodox	99.6	82.8	58.6	44.1	−16.8	−24.2	−14.5	−55.5
Catholic	107.5	39.2	104.5	57.7	−68.3	65.3	−46.8	−48
Protestant	82.7	73.5	60.4	43.8	−9.2	−13.1	−16.6	−38.9
Muslim	105.7	83.4	58.9	53.7	−22.3	−24.5	−5.2	−52
Others	97.0	50.9	65.9	38.9	−46.1	15	−27	−58.1
**Age of women (in years)**								
< 20	137.7	121.5	93.3	47.7	−16.2	−28.2	−45.6	−90
20–29	101.5	82.1	57.9	45.4	−19..4	−24.2	−12.7	−56.1
30–39	83.6	68.3	55.1	54.6	−15.3	−13.2	−0.5	−29
≥ 40	117.5	89.2	72.5	46.9	−28.3	−16.7	−25.6	−70.6
**Maternal education**								
No education	102.6	83.9	63.2	51.1	−18.7	−20.7	− 12.1	−51.5
Primary	83.8	71.4	54.5	42.2	−12.4	−16.9	− 12.3	−41.6
Secondary and higher	73.5	38.1	32.5	40.5	−35.4	−5.6	8	−33
**Husband education**								
No	104.6	88.4	55.3	51.1	−16.2	−33.1	−4.2	−53.5
Primary	93.3	74.4	40.4	32.0	−18.9	−34	−8.4	−61.3
Secondary and higher	78.8	51.7	64.8	48.1	−27.1	−13.1	− 16.7	−30.7
**Sex of household head**								
Male	98.4	78.9	60.5	48.9	−19.5	− 18.4	−11.6	−49.5
Female	101.0	87.6	54.8	42.6	−13.4	−32.8	−12.2	−57.4
**Maternal BMI (in Kg/m**^**2**^**)**								
< 18.5	99.0	76.6	68.6	55.2	−22.4	−8	−13.4	−43.8
18.5–24.9	97.7	77.7	56.7	46.4	−20	− 21	−10.3	−51.3
≥ 25	120.0	82.1	64.9	46.3	−37.9	−17.2	− 18.6	−73.7
**Number of ANC visit during pregnancy**								
No visit	66.4	57.8	40.6	47.7	−8.6	−17.2	7.1	−18.7
1–4 visits	73.9	57.3	42.5	31.3	−16.6	−14.8	− 11.2	−42.6
> 4 visits	33.7	34.4	35.4	20.5	0.7	1	−14.9	−13.2
**Size at birth**								
Small	92.2	91.8	57.1	56.8	−0.4	−34.7	− 0.3	−35.4
Average	85.4	69.6	45.4	41.0	−15.8	−24.2	−4.4	−44.4
Large	121.3	81.1	79.1	49.9	−40.2	−2.0	−29.2	−71.4
**Place of delivery**								
Home	98.1	78.2	58.8	49.8	−19.9	− 19.4	−9.0	−48.3
Health facility	109.4	106.4	67.0	43.3	−3.0	−39.4	−23.7	−66.1
**Mode of delivery**								
Vaginal	98.9	79.8	59.5	47.4	−19.1	−20.3	−12.1	−51.5
Caesarean section	77.9	93.0	74.6	82.8	15.1	−18.4	8.2	4.9
**Type of birth**								
Single	90.3	76.6	55.6	44.0	−13.7	−21.0	−11.6	−46.3
Multiple	360.9	260.0	167.3	196.2	−100.9	−92.7	28.9	− 164.7
**Sex of infant**								
Male	104.8	92.2	69.6	59.9	−12.6	−22.6	−9.7	−44.9
Female	92.3	66.9	49.0	35.2	−25.4	−17.9	− 13.8	−57.1
**Ever had termination of pregnancy**								
No	99.2	80.0	60.6	47.2	−19.2	− 19.4	− 13.4	−52.0
Yes	95.7	79.2	52.1	57.1	−16.0	−27.1	−5.0	−38.6
**Stunting**								
Normal	0	0	0	0	0	0	0	0
moderately stunted	0	0	0	0	0	0	0	0
Severely stunted	158	97.5	260.4	204.0	−60.5	162.9	−56.4	46.0
**Underweight**								
Normal	0	0	0	0	0	0	0	0
Moderately underweight	0	0	0	0	0	0	0	0
Severely underweight	153	94.0	359.9	281.0	−69	265.9	−78.9	128
**Wasting**								
Normal	0	0	0	0	0	0	0	0
Moderately wasted	0	0	0	0	0	0	0	0
Severely wasted	109	83.0	581.5	376.7	−26	498.5	− 204.8	267.7
**Media exposure**								
No	102.1	80.6	59.1	42.9	−21.5	−21.5	−16.2	−60.2
Yes	89.6	78.8	60.1	58.5	−10.8	− 18.7	− 1.6	−31.1
**Preceding birth interval**								
< 24 months	150.4	131.5	94.1	83.6	−18.9	−37.4	− 10.5	−66.8
≥ 24 months	88.9	66.9	52.9	40.4	−22.0	−14.0	−12.5	−48.5
**Parity**								
1–3	99.1	77.6	52.8	43.9	−21.5	−24.8	−8.9	−55.2
4–6	87.5	72.7	64.2	48.3	−14.8	−8.5	−15.9	−39.4
> 6	113.7	94.7	91.0	80.9	−19.0	−3.7	−10.2	−34.8

### Decomposition analysis

There was a significant decline in the infant mortality rate for the last 16 years (2000–2016). The overall multivariate decomposition analysis revealed that about 18.1% of the overall decrease in infant mortality rate over the last 16 years was attributable to the difference in endowment (composition) across the surveys whereas the remaining 81.9% was attributable to the difference in coefficient (effects of characteristics) over the surveys (Table 3). Among the difference in endowment; the difference in composition of rural residents (*B = − 0.0006, 95% CI: − 0.0009, − 0.0004, p = 0.03*), maternal age 30–39 years (*B = 0.0007, 95% CI: − 0.001, − 0.0002, p = 0.001*), 40–49 years (*B = 0.0008, 95% CI: 0.0002, 0.0014, p = 0.04*), multiple births (*B = − 0.0003, 95% CI: − 0.0002, − 0.0004, p = 0.003*), and births from mother who have > 6 births (*B = − 0.005, 95% CI:-0.0086, − 0.0018, p = 0.02*) were significantly contributed for the decrease in infant mortality rate over the last 16 years. Among the difference in coefficients, the difference in effects of rural residents (*B = − 0.02, 95% CI: − 0.0001, − 0.047, p = 0.003*), multiple births (*B = − 0.0008, 95% CI: − 0.0015, − 0.0018, p = 0.012*), health facility delivery (*B = 0.004, 95% CI: 0.001, 0.006, p = 0.023*), and husband with secondary education or higher (*B = 0.0036, 95% CI: 0.0002, 0.007, p = 0.001*) were significantly contributed to the decrease in infant mortality rate over the last 16 years (Table 4).

**Table 3:** The overall Logit based multivariate decomposition analysis of infant mortality in Ethiopia, 2000–2016

Infant mortality	Coef.	[95% Conf. Interval]	Pct.
E	−0.008	−0.016 -0.0007	18.1^a^
C	−0.037	− 0.047 -0.027	81.9^a^
R	−0.045	− 0.052 -0.038	

^a^*E* Endowment, *C* Coefficient, *R* Residuals, *Coef* Coefficient, *Pct* Percent

**Table 4:** The detailed Logit based multivariate decomposition analysis of infant mortality in Ethiopia, 2000–2016

Infant death	Difference due to characteristics(E)		Difference due to coefficient (C)	
	Coef. with *p*-value	Pct.	Coef. with *p*-value	Pct.
**Residence**				
Urban	0		0	
Rural	− 0.0006[− 0.0009, − 0.0004]^*^, 0.03	−1.08	− 0.02 [− 0.0001, − 0.047]^**^, 0.003	− 52.1
**Age of women**				
< 20	0		0	
20–29	−0.0001 [− 0.0003, 0.0007], 0.23	0.29	0.005 [− 0.013, 0.022], 0.12	− 10.4
30–39	− 0.0007 [− 0.001, − 0.0002]^**^, 0.001	1.4	0.004 [− 0.01, 0.018], 0.06	−8.3
≥ 40	0.0008 [0.0002, 0.0014]^*^, 0.04	−1.8	−0.001 [− 0.006, 0.004], 0.21	2.4
**Maternal education**				
No education	0		0	
Primary	−0.0006 [− 0.002, 0.0008], 0.07	1.3	− 0.0002[− 0.003, 0.003], 0.42	0.05
Secondary and higher	− 0.0003 [− 0.001, 0.0004], 0.31	0.71	0.0017 [− 0.001, 0.004], 0.34	1.9
**Type of birth**				
Single	0		0	
Multiple	−0.0003 [− 0.0002, − 0.0004]**, 0.003	−0.69	− 0.0008 [− 0.0015, 0.0018]*, 0.012	−1.9
**Place of delivery**				
Home	0		0	
Health facility	−0.002 [− 0.005, 0.0006], 0.08	4.4	0.004 [0.001, 0.006]*, 0.023	−7.9
**Parity**				
1–3	0		0	
4–6	0.00005[−0.0003, 0.0003], 0.09	−0.3	0.005 [− 0.002, 0.011], 0.07	− 10.1
> 6	− 0.005 [− 0.0086, − 0.0018]*, 0.02	11.5	0.001 [− 0.002, 0.004], 0.09	−16.4
**Ever had termination of pregnancy**				
No	0		0	
Yes	−0.0005 [− 0.0014, 0.0004], 0.071	1.07	0.0014 [− 0.0022, 0.005], 0.51	−3.06
**Body mass index of women (kg/m**^**2**^**)**				
< 18.5	0		0	
18.5–24.9	0.0006 [−0.0002, 0.001], 0.4	−1.36	−0.014 [− 0.026, 0.0001], 0.21	31.4
≥25	−0.0014 [− 0.002, 0.0003], 0.37	2.33	−0.0014 [− 0.003, 0.0001], 0.17	3.06
**Husband education**				
no education	0		0	
Primary	0.0003 [−0.0017, 0.0024], 0.08	−0.74	0.0005 [− 0.0055, 0.0065], 0.47	−1.12
Secondary	0.0008 [−0.0035, 0.0052], 0.13	−1.8	0.0036 [0.0002, 0.007]^**^, 0.001	−8.03
**Media exposure**				
No	0		0	
Yes	0.0001 [−0.0007, 0.0003], 0.19	−0.28	0.0039 [− 0.0018, 0.0097], 0.07	− 8.8
**Constant**			−0.071 [− 0.119, − 0.023], 0.3	157.14

### Determinants of infant mortality

#### Model comparison

The mixed-effect binary logistic regression model was the best-fitted model since it had a lower deviance value (Table 5). The ICC value was 0.13(95% CI: 0.09, 0.21), which indicates that about 13% of the overall variability of infant mortality was due to the between cluster variability, and the LR test was (X^2^ = 10.44, *p* = 0.0006) which informed us the mixed-effect binary logistic regression model (GLMM) was the best-fitted model. Moreover, the MOR-value in the null model was 1.98 (95% CI: 1.81, 2.21), indicates that infants in high infant mortality clusters were a 1.98 times higher likelihood of dying in their first year of life compared to infants in low infant mortality clusters. The PCV value in the final model was 0.13, which showed that the final model explained the variability in infant mortality by the final model was 13% (Table 6).

**Table 5:** Model comparison between standard logistic regression and mixed-effects logistic regression

Model comparison	AIC	BIC	Deviance
Logistic regression model	4033.79	4221.50	3987.80
Mixed effect logistic regression model	4026.93	4201.47	3978.94

*AIC* Akaike Information Criteria, *BIC* Bayesian Information Criteria

**Table 6:** Random effect parameters

Random effect parameters	Null model	Full model
Cluster variance	0.52 (0.39, 0.70)	0.45 (0.23, 0.91)
ICC	0.1 (0.04, 0.13)	0.06 (0.015, 0.20)
MOR	1.98 (1.81, 2.21)	1.89 (1.58, 2.48)
PCV	Ref.	0.13

*ICC* Intra-class Correlation Coefficient, *MOR* Median Odds Ratio, *PCV* Proportional Change in Variance

In the multivariable mixed-effect logistic regression model; size at birth, preceding birth interval, the number of ANC visits, maternal age, type of birth, maternal BMI, sex of the child, and parity were significant determinants factors of infant mortality. The odds of mortality among infant born to mothers aged 20–29 years, 30–39 years, and 40–49 years were decreased by 37% (95% CI: 0.41, 0.99, *p = 0.02*), 48% (95% CI: 0.32, 0.84, *p = 0.001*), and 49% (95% CI: 0.28, 0.93, p *= 0.02*) compared to those born to mothers aged < 20 years, respectively. Infants born to mothers having the preceding birth interval of < 24 months were 1.79 (95 CI: 1.46, 2.19, *p = 0.05*) times higher odds of death within the first year of birth than those born with a preceding birth interval of 24 months or above. The odds of infant mortality among infants who were small and large size at birth were 1.55 (95% CI: 1.25, 1.92, *p = 0.006*) and 1.26 (95% CI: 1.01, 1.57, *p = 0.003*) times higher than those infants who were average size at birth, respectively. The odds of mortality among infants born from underweight women (< 18.5 kg/m^2^) were 1.22 (95% CI: 1.05, 1.50, *p = 0.03*) times higher than those born to mothers with normal BMI (18.5–24.9 Kg/m^2^). Births from mothers who had no ANC visits during pregnancy had a 3.14 (95% CI: 2.11, 4.66, *p = 0.001*) times higher likelihood of death within the first year of birth than an infant born to mothers who had > four ANC visit during pregnancy. The odds of infant death among twins were 4.25 (95% CI: 3.01, 6.01, *p = 0.0001*) times higher than those in single births. The infant born to multiparous women having greater than 6 children had a 1.51 (95% CI: 1.01, 2.26, *p = 0.01*) times higher odds of death than compared to an infant born to women having 1–3 births. Being male had a 1.50 (95% CI: 1.25, 1.79, *p = 0.007*) higher odds of death in the first year of life as compared to female infants (Table 7).

**Table 7:** The bi-variable and multivariable mixed-effect logistic regression analysis of determinants of infant mortality among births from reproductive-age women in Ethiopia, 2016

Variables	Infant status		Crude Odds Ratio (COR) with 95% CI	***p***-value	Adjusted Odds Ratio (AOR) with 95% CI	***p***-value
	Alive	Died				
**Residence**						
Urban	1913	61	1		1	
Rural	8187	480	1.85 [1.39, 2.48]	0.001	1.42 [0.98, 2.06]	0.19
**Sex of household head**						
Male	7944	439	1		1	
Female	2156	102	0.85 [0.68, 1.07]	0.16	0.89 [0.70, 1.12]	0.68
**Wealth status**						
Poor	5441	334	1.42 [1.14, 1.75]	0.002	0.84 [0.65, 1.09]	0.58
Middle	1399	67	1.12 [0.82, 1.51]	0.51	0.81 [0.58, 1.13]	0.19
Rich	3260	140	1		1	
**Parity**						
1–3	6003	291	1		1	
4–6	3457	193	1.14 [0.95, 1.39]	0.16	1.01 [0.79, 1.29]	0.93
> 6	640	57	1.82 [1.34, 2.47]	0.0001	1.51 [1.01, 2.26]^*^	0.01
**Maternal BMI (Kg/m**^**2**^**)**						
< 18.5	2306	153	1.27 [1.04, 1.56]	0.001	1.22 [1.05, 1.50]^*^	0.03
≥25	1306	50	0.73 [0.54, 0.99]	0.05	0.78 [0.57, 1.08]	0.44
18.5–24.9	6488	338	1		1	
**Covered by health insurance**						
Yes	316	5	1		1	
No	9784	536	3.28 [1.33, 8.06]	0.01	2.49 [0.99, 6.18]	0.25
**Sex of the child**						
Male	5157	326	1.44 [1.21, 1.72]	0.001	1.50 [1.25, 1.79] ^**^	0.007
Female	4943	215	1		1	
**Type of birth**						
Single	9875	488	1		1	
Multiple	223	53	5.01 [3.60, 6.97]	0.0001	4.25 [3.01, 6.01] ^**^	0.0001
**Place of delivery**						
Health facility	3345	141	1		1	
Home	6755	400	1.37 [1.11, 1.68]	0.03	0.78 [0.61, 1.05]	0.83
**Maternal age (in years)**						
< 20	379	25	1		1	
20–29	5054	278	0.84 [0.55, 1.29]	0.21	0.63 [0.41, 0.99]^*^	0.02
30–39	3864	193	0.76 [0.49, 1.18]	0.19	0.52 [0.32, 0.84]^**^	0.001
≥40	803	45	0.85 [0.51, 1.43]	0.2	0.51 [0.28, 0.93]^*^	0.02
**Maternal education**						
No	6460	378	1.57 [1.11, 2.22]	0.01	0.96 [0.64, 1.44]	0.73
Primary	2554	124	1.33 [0.92, 1.94]	0.13	1.01 [0.67, 1.51]	0.61
Secondary and higher	1086	39	1		1	
**Number of ANC visit during pregnancy**						
No	5506	423	3.50 [2.42, 5.07]	0.0001	3.14 [2.11, 4.66] ^*^	0.001
1–4	3146	86	1.23 [0.81, 1.86]	0.32	1.14 [0.75, 1.74]	0.65
> 4	1452	32	1		1	
**Preceding birth interval**						
< 24 months	1943	175	1.97 [1.63, 2.38]	0.001	1.79 [1.46, 2.19]^*^	0.05
≥24 months	8157	366	1		1	
**Size at birth**						
Average	3051	163	1		1	
Small	2811	197	1.64 [1.32, 2.02]	0.0001	1.55 [1.25, 1.92]^**^	0.003
Large	4238	181	1.24 [0.99, 1.55]	0.015	1.26 [1.01, 1.57]^**^	0.006

^*^ANC Antenatal Care Utilization, *AOR* Adjusted Odds Ratio, *BMI* Body Mass Index, *CI* Confidence Interval, *COR* Crude Odds Ratio, *p-value < 0.05, **p-value < 0.01

## Discussion

The trends of infant mortality in Ethiopia was decreased from 96.9 to 48 per 1000 live births with an annual rate of reduction of 4.2%. This was consistent with a study reported in sub-Saharan Africa [34]. This might be because of improvement in the management of childhood illness over time [35], increased universal immunization coverage mainly targeting the commonest cause of infant mortality such as pneumonia, diarrheal diseases (Rotavirus), pertussis, and measles [36]. Besides, in the last two decades, the establishment of health extension workers plays a significant role in improving maternal and child health by providing preventive (such as a vaccine, ANC service) services and extending services to rural residents [31, 37]; possibly contributing to the significant decrease in the infant mortality rate for the last 16 years in Ethiopia.

The multivariate decomposition analysis identified the significant factors that contributed to the decrease in infant mortality rate over the last 16 years. The overall decrease in infant mortality rate over the last 16 years was associated with the difference in the composition of rural residents, maternal age, parity and type of birth, and the difference in effects of rural residents, health facility delivery, and husband education. This was supported by the study findings reported in Indonesia [32], Nicaragua [18, 38], Nigeria [18, 19], and Canada [39]. This could be **because** teenage pregnancies are more likely to suffer from adverse pregnancy outcomes both during and after birth including anemia, asphyxia, low birth weight, Intra-uterine Growth Reduction (IUGR), and congenital malformation; therefore the decrease in teenage pregnancy over time may contribute to the reduction of infant mortality [40]. Ethiopia proposed maternal education to increase a mother’s knowledge of health care practices related to contraceptive utilization, nutrition, hygiene, and disease prevention in the reduction of infant mortality [34]. Furthermore, improved services provision at health facilities for women and the availability of skilled professionals for saving maternal and newborn lives.

In the mixed effect logistic regression analysis; size at birth, preceding birth interval, the number of ANC visits, maternal age, type of birth, sex of the child, maternal BMI, and parity were significant predictors of infant mortality. In this study, child size at birth was a significant predictor of infant mortality. The infant who was small or large size at birth were significantly associated with higher odds of infant mortality than average size infants. This was consistent with study findings in the USA [41] and Bangladesh [42]. This might be due to small size babies are commonly due to preterm birth or small for gestational age, they are prone to sepsis, hypothermia, and undernutrition and this could increase their risk of mortality. Also, low birth weight babies might have underlined medical conditions such as congenital heart diseases, down syndrome, HIV/AIDS, or other diseases and this could make them vulnerable to childhood infections like pneumonia and diarrheal diseases, consequently increase their risk of death. Regarding large size babies, commonly macrosomia is a result of maternal underlined diseases like diabetic mellites, chronic illness, and genetic causes, this could increase their risk of mortality before reaching the first year of age [43]. The shorter preceding birth interval was significantly associated with higher odds of infant mortality. This was consistent with the study findings in Malawi [44] and Zimbabwe [45]. The possible explanation might be since shorter preceding birth intervals are associated with increased risk of preterm birth, low birth weight, and IUGR for the succeeding births [46]. Besides, mothers had less time to recuperate from the previous birth, and less able to provide nourishment for the infant, this might increase the risk of infant mortality [47].

Babies born to underweight mothers (BMI <  18.5Kg/m^2^) have higher odds of death in the first year of birth. This finding is consistent with previous study findings [48]; malnourished mothers have an increased risk of poor pregnancy outcomes including obstructed labor, premature delivery, and low-birth-weight babies [49]. Therefore, an infant born to a malnourished mother is more prone to malnutrition and childhood illnesses like diarrheal diseases and respiratory diseases which are the leading cause of child mortality [50]. In this study, parity and twin birth were significant predictors of infant mortality; an infant born to multiparous women who have greater than 6 births, and twin births, had higher odds of death before reaching the first year of birth. This was consistent with studies reported in Pristina [51] and Australia [52]. The possible reason could be due to multiparous women and twins are related to an increased rate of adverse perinatal outcomes, such as premature birth and low birth weight [53]. The higher mortality rate among multiple births compared with single births could be because twins are more likely to be born prematurely, higher risk of malnutrition, and more likely to be of lower birth weight than single infants this could increase the risk of death within 1 year of birth [54].

In our study, ANC was a significant predictor of infant mortality. The odds of infant mortality among women who had no ANC visit during pregnancy was higher than those who have ANC checkup. This was supported by the study findings in India [55] and Nepal [56], it could be due to reason that ANC visit is an entry point for the other maternal health services, and births from mother who had no ANC visit are not aware of danger signs of pregnancy and underlying medical conditions that could lead to low birth weight, prematurity, congenital anomalies as compared to women who had ANC visit [57]. The odds of infant mortality among births mothers aged ≥20 years were lower than births from mothers aged less than 20 years. It was consistent with prior studies [58], the possible explanation could be due to the reason that teenagers have biological immaturity and nutrition which could increase the risk of infant mortality [59]. Besides, teenagers are less likely to use maternal health care services such as ANC, institutional delivery, PNC, and routine immunization this could increase the odds of infant mortality [60]. Male infants were significantly associated with higher odds of infant mortality, which is supported by previous studies [61, 62]. The possible explanation for this difference might be due to sex differences in genetic and biological makeup, with boys being biologically weaker and more susceptible to diseases and premature death [63].

## Strength and limitations

This study had several strengths. First, the study was based on nationally representative large datasets, and thus it had adequate statistical power. Second, the estimates of the study were done after the data were weighted for the probability sampling and non-response, to make it representative at national and regional levels: therefore, it can be generalized to all births from reproductive-age women in Ethiopia. Third, multivariate decomposition analysis was applied to understand the factors that significantly contributed to the decrease in infant mortality over time. Limitations included that variables were not consistently collected in all EDHS surveys; the wealth index was not collected in EDHS 2000 even if it was planned for collection, thus this variable was not used for the decomposition analysis. In addition, important variables such as underlying medical conditions such as pneumonia, meningitis, birth asphyxia, congenital heart diseases, diarrheal diseases, sepsis, HIV/AIDS, congenital infections were not considered in the model as these variables didn’t found in the EDHS. Furthermore, the EDHS survey did not incorporate community-level variables like community norm, culture, and beliefs, and medical factors rather it relied on mothers or caregivers report and might have the possibility of social desirability and recall bias since infant mortality is not socially acceptable though CSA claims that strong effort was made to minimize it mainly through extensive training of data collectors, recruiting experienced data collectors and supervisors this might underestimate our finding.

### Policy implications of this study

Infant mortality has been considered as the crucial indicator of the quality of the health care delivery system and progress of Ethiopia. Though the infant mortality rate in Ethiopia showed a significant reduction over time, still we are expected to have double progress to achieve the ENAP plan. To keep this progress maternal and child health programs should focus on promoting ANC visits, and adequate birth spacing as these factors are amenable to change. Moreover, health care providers should give special attention to abnormal weight babies, multiple births, and male births to reduce the incidence of infant mortality in the country.

## Conclusions

The infant mortality rate has shown a dramatic decrease over the last 16 years in Ethiopia. The multivariate decomposition analysis revealed that about 18.1% of the overall decrease in infant mortality was attributable to the difference in endowment (the composition of respondents) in terms of residence, maternal age, type of birth, and parity across the surveys whereas the remaining 81.9% was due to the difference in the effect of residence, parity, health facility delivery and husband education over the surveys. These findings highlight that the governmental and non-governmental organizations should scale up health facility delivery and give special attention to twin births, multiparous women, rural dwellers to further reduce the infant mortality rate in Ethiopia. Besides; birth interval, maternal BMI, size at birth, parity, maternal age, type of birth, sex of the child, and ANC visit were significantly associated with infant mortality. Therefore, it could help the policymakers and health planners to focus on designing prevention programs and health care delivery, thus allocating public health resources for further reducing infant mortality.

## Data Availability

Data is available online and you can access it from www.measuredhs.com.

## Abbreviations

ANC: Antenatal Care
AOR: Adjusted Odds Ratio
COR: Crude Odds Ratio, CSA=Central Statistical Agency
DHS: Demographic health survey
EAs: Enumeration areas
EDHS: Ethiopian demographic and health survey
GIS: Geographic Information System
ICC: Intra-cluster Correlation Coefficient
IUGR: Intra uterine growth restriction
LLR: Log likelihood ratio
LR: Likelihood ratio
MDG: Millennium Development Goals
RR: Relative risk
SNNP: Southern Nations and Nationalities of People
